# Factors associated with suicide/self-inflicted injuries among women aged 18–65 years in the United States: A 13-year retrospective analysis of the National Inpatient Sample database

**DOI:** 10.1371/journal.pone.0287141

**Published:** 2023-10-03

**Authors:** Oluwasegun Akinyemi, Temitope Ogundare, Adeolu Funsho Oladunjoye, Kindha Elleissy Nasef, Christina Lipscombe, John Akinshola Akinbote, Maureen Bezold

**Affiliations:** 1 Clive O Callender Department of Surgery, Howard University, Washington, D.C., United States of America; 2 Department of Psychiatry, Boston Medical Center, Boston, MA, United States of America; 3 Department of Psychiatry, Boston University School of Medicine, Boston, MA, United States of America; 4 Department of Obstetrics and Gynecology, Howard University, Washington, D.C., United States of America; 5 Department of Health Sciences and Social Work, Western Illinois University, Macomb, Illinois, United States of America; Dilla University, ETHIOPIA

## Abstract

**Background:**

Suicide is a significant cause of mortality in the United States, accounting for 14.5 deaths/100,000. Although there are data on gender disparity in suicide/self-inflicted injury rates in the United States, few studies have examined the factors associated with suicide/self-inflicted injury in females.

**Objective:**

To determine factors associated with suicide/self-inflicted injuries among women aged 18–65 years in the United States.

**Methods:**

Hospitalizations for suicide or self-inflicted injuries were identified using the National Inpatient Sample database from 2003–2015 using sample weights to generate national estimates. Independent predictors of suicide/self-inflicted injuries were identified using multivariable regression models. Interaction term analysis to identify the interaction between race/ethnicity and income were conducted.

**Results:**

There were 1,031,693 adult women hospitalizations in the U.S. with a primary diagnosis of suicide/self-inflicted injury in the study period. The highest suicide/self-inflicted injury risk was among women aged 31-45years (OR = 1.23, CI = 1.19–1.27, p < 0.05). Blacks in the highest income strata had a 20% increase in the odds of suicide/self-inflicted injury compared to Whites in the lowest socioeconomic strata (OR = 1.20, CI = 1.05–1.37, p <0.05). Intimate partner violence increased suicide/self-inflicted injury risk 6-fold (OR = 5.77, CI = 5.01–6.65, p < 0.05).

**Conclusion:**

Suicide risk is among women aged 31–45 years, higher earning Black women, intimate partner violence victims, uninsured, and current smokers. Interventions and policies that reduce smoking, prevents intimate partner violence, addresses racial discrimination and bias, and provides universal health coverage are needed to prevent excess mortality from suicide deaths.

## Introduction

Suicide is a significant cause of mortality in the United States, accounting for 14.5 deaths per 100,000 population [1]. It is the second leading cause of death among individuals aged 10 and 34 years and the fourth leading cause of death for individuals aged 35–44 years in the United States [1]. Suicide attempts and self-inflicted injuries represent a major risk factor for completed suicides [2, 3]. In 2020, over 1.2 million people over the age of 18 reported a suicide attempt in the United States and hundreds of thousands more presenting to hospitals with self-harm injuries [4]. Suicide attempts can be defined as a non-fatal self-directed potentially injurious behavior with any intent to die as a result of the behavior; although self-inflicted injuries often include non-suicidal intent, it remains a powerful predictor of suicide [5].

Since 1999, there has been a significant increase in suicide/self-inflicted injuries rates with a disproportionate increase in females compared to males in the United States [6]. Women are three times more likely to attempt suicide, while men are more likely to complete suicide [7]. According to a study by Curtin and colleagues [8], from 2000 to 2014, the age-adjusted suicide rate among women in the United States increased from 4.0 to 5.8 per 100,000. In 2017, the suicide rate was the highest among non-Hispanic white females aged 45–64 years (12.8/100,00) and non-Hispanic American Indian/Alaskan Native females aged 25–44 years (20.7/100,00) in the United States [9]. Traditionally, suicide research has focused on the mortality of suicidal behaviors, which for the most parts favor males, who tend to die 2–4 times more than females; and have often viewed suicide attempts in females as manipulative or inept, overlooking the clear suicidal intent behind many of these suicide attempts [10–12]. However, when mortality and morbidity of suicide are considered, females contribute far more to the burden [10]. Therefore, more research on suicidal behaviors in women is warranted.

Women’s greater vulnerability to suicidal behaviors have been explained in part by a greater vulnerability to psychopathology and psychosocial stressors [10]. Regarding psychopathology, previous studies have found associations between depressive disorders, anxiety disorders, borderline personality disorder, eating disorders, PTSD, antisocial personality disorders, history of violence, substance use disorder and suicide [13–19]. Except for antisocial personality disorders, and substance use disorders, women report higher rates of these mental disorders [20–22]. In addition, other psychosocial factors associated with suicide include: being a victim of domestic violence; physical, emotional, and sexual abuse; lower levels of education, being single, and financial instability [10, 13, 23–25]. Women are more likely to be victims of domestic abuse, more likely to have experienced abuse (including physical, emotional, and sexual abuse), and more likely to have less education and lower socioeconomic status compared to males [10, 26–28]. In addition, marriage, which is deemed protective for males, has not been shown to be protective for females [10, 29]. Domestic violence and family dysfunction have been shown to predict suicidality in females but not in males [24].

There is fewer research on suicide risk factors in the general population compared to high-risk populations, and even fewer specific to women [10, 23, 30, 31]. About 33% of suicide attempts occur in people who have no prior contact with mental health services, making identification of other ecological factors that increase risk of suicide imperative [10, 23]. This study aims to determine factors associated with suicide/self-inflicted injury among women aged 18–65 years in the United States over 12 years, from 2003–2015.

## Methods

This is a retrospective analysis of all hospitalizations with the diagnosis of self-injury or attempted suicide/ in the National Inpatient Sample (NIS) database (2003–2015). The NIS is the largest all-payer inpatient database in the United States of America and comprises a 20% stratified random sample of all U.S. hospital discharges. Each of these discharges in the NIS is de-identified. Further details on the NIS design are available at. https://www.hcup-us.ahrq.gov/. The NIS was redesigned in 2012 to improve the prediction of national estimates. To account for this, we used trend weight (TRENDWT) for 2003–2011 and the regular discharge weight (DISCWT) for 2012–2015. From 150,819,065 hospitalizations in the NIS between 2003 and September 2015, we excluded all hospitalizations in the 4th quarter of 2015. This was done to remove the impact of transitioning from the International Classification of Diseases—ninth edition (ICD-9) to the International Classification of Diseases—tenth edition (ICD- 10) codes on October 1st, 2015. We also excluded hospitalizations with ages < 18 or > 65yrs and all hospitalizations with missing variables.

Patient’s age was stratified as 18-30yrs, 31-45yrs and 46-65yrs; race/ethnicity as White, Black, Hispanics, and Others; and insurance status as Private, Medicare, Medicaid, Uninsured, and Others. We utilized the NIS annual median income generated from the zip codes and subsequently grouped them into quartiles each year by Healthcare Cost and Utilization Project (HCUP). This was stratified into four quartiles, with Quartile 1 corresponding to the lowest median income quartile and quartile 4, the highest. Patients’ smoking habits were classified as non-smokers, previous smokers, and current smokers. Intimate partner violence was defined using the International Classification of Diseases, Clinical Modification (ICD-CM) codes and the ICD-CM Supplementary Classification of External Causes of Injury and Poisoning Codes (E-codes) 995.80–995.85, V7181 and E967.3).

### Main outcome

The study’s primary outcome was the occurrence of suicide or self-inflicted injury. We identified the ICD-CM supplementary classification of external causes of injury and poisoning codes (E codes) for suicide or self-inflicted injury with E950-E959 diagnostic codes. These codes have a positive predictive value of 83–100% in the literature [32].

### Statistical analysis

Descriptive statistics such as frequencies and percentages describe patients’ baseline characteristics and risk factor variables. Our bivariate analysis utilized Pearson chi-square tests to evaluate the relationship between studied variables and the occurrence of suicide or self-inflicted injury. We included statistically significant variables in the final multivariate regression analyses and estimated risk of suicide or self-inflicted injury as adjusted odds ratios and 95% confidence intervals. The multivariate regression analyses also explored the interaction between race/ethnicity and median income by creating an interaction term, race × median income. A 2-tailed p-value <0.05 was considered statistically significant. All statistical analyses were performed using the STATA 14 (Stata Corp College Station, TX).

### Ethics

The study was conducted in accordance with the ethical standards of the institutional and/or national research committee and with the 1964 Helsinki declaration and its later amendments or comparable ethical standards. Institutional Review Board approval was waived because the study was carried out on a national database that contained de-identified data and did not require informed consent or direct participation of patients.

## Results

We identified 1,031,693 hospitalizations of adult women with a primary diagnosis of suicide or self-inflicted injury between January 2003 and September 2015. Table 1 shows the demographic distribution of these patients by race. Among the study population, more White women (31.1%) were aged 46–65 years, compared to Blacks (22.6%), Hispanics (22.2%), and women identifying as Others (23.3%). Regarding income, more Black women (49.1%) were in the lowest income quartile compared to Hispanics (40.3%), Others (28.9%), and White (24.8%). Women identifying as Hispanics (19.0%) had the highest uninsured rate compared to Blacks (17.2%), and Whites (16.2%). Smoking habits also differed by race, with 28.8% of Whites being current smokers compared to 22.7% of Blacks and 17.6% of Hispanics (p<0.001). There was no racial/ethnic difference in the experience of intimate partner violence.

**Table 1:** Sociodemographic characteristics of women aged 18–65 years with suicide/self-inflicted injury between 2003–2015.

Variables	White	Black	Hispanic	Others	p-value
**Age (%)**					**<0.001**
18-30yrs	28.8	36.8	37.1	37.6	
31-45yrs	40.1	40.6	40.7	39.1	
46-65yrs	31.1	22.6	22.2	23.3	
**Median Income (%)**					**<0.001**
Lowest Quartile	24.8	49.1	40.3	28.9	
2nd Quartile	27.7	21.3	24.3	22.6	
3rd Quartile	25.8	18.0	22.1	23.1	
Highest Quartile	21.8	11.6	13.3	25.4	
**Insurance (%)**					**<0.001**
Private	38.5	22.4	27.6	36.8	
Medicare	15.1	12.8	8.8	9.1	
Medicaid	23.9	41.2	36.2	28.1	
Uninsured	16.2	17.2	19.0	18.2	
Others	6.3	6.4	8.4	7.8	
**Smoking (%)**					**<0.001**
Non-smokers	70.1	76.3	81.6	79.9	
Former smokers	1.1	0.9	0.8	0.8	
Current smokers	28.8	22.7	17.6	19.3	
**Intimate Partner Violence (%)**	0.2	0.2	0.3	0.3	0.32

In the bivariate analysis (Table 2), suicide/self-inflicted injury was more common among women aged 31–45 years (p<0.001), Whites (p<0.001), and those with private insurance (p<0.001). In the multivariate analysis (Table 3), the independent predictors of suicide/self-inflicted injury were: age, race, intimate partner violence, smoking, and insurance type.

**Table 2:** Correlates of suicide/self-inflicted injury among women aged 18–65 years between 2003–2015.

Variables	Suicide/self-inflicted injury		p-value
	Yes (%)	No (%)	
**Age**			**<0.001**
18-30yrs	31.2	31.9	
31-45yrs	40.3	30.0	
46-65yrs	28.5	38.1	
**Race**			**<0.001**
White	78.6	60.4	
Black	9.0	17.1	
Hispanics	7.4	15.4	
Others	5.0	7.2	
**Median Income**			**<0.001**
Lowest Quartile	28.3	29.1	
2nd Quartile	27.7	25.7	
3rd Quartile	24.7	23.8	
Highest Quartile	19.3	21.3	
**Insurance**			**<0.001**
Private	36.3	50.0	
Medicare	14.0	11.9	
Medicaid	26.9	27.6	
Uninsured	16.4	6.2	
Others	6.5	4.4	
**Smoking**			**<0.001**
Non-smokers	72.0	85.8	
Former smokers	1.0	3.1	
Current smokers	27.0	11.1	
**Intimate Partner Violence**	0.2	99.8	**<0.001**

**Table 3:** Predictors of suicide/self-inflicted injury among women ages 18-65yrs (NIS 2003–2015).

Variables	Odds Ratio	p-value	95% CI
**Age groups**			
18–30	Reference		
31–45	1.23	<0.0001	1.19–1.27
46–65	0.61	<0.0001	0.59–0.63
**Race**			
Whites	Reference		
Blacks	0.36	<0.0001	0.33–0.38
Hispanics	0.34	<0.0001	0.30–0.39
Others	0.60	<0.0001	0.54–0.66
**Income**			
Lowest Quartile	Reference		
Quartile 2	1.04	0.18	0.98–1.09
Quartile 3	1.05	0.17	0.98–1.11
Quartile 4	1.01	0.87	0.93–1.09
**Smoking**			
Non-smokers	Reference		
Former smokers	0.41	<0.0001	0.38–0.44
Current smokers	2.33	<0.0001	2.24–2.43
**Race x Income**			
White x Lowest Income	Reference		
Black x Quartile 2	0.95	0.22	0.88–1.03
Black x Quartile 3	1.12	0.10	0.98–1.28
Black x Quartile 4	1.20	**0.01**	1.05–1.37
Hispanics x Quartile 2	0.96	0.54	0.83–1.10
Hispanics x Quartile 3	1.01	0.87	0.87–1.19
Hispanics x Quartile 4	1.01	0.86	0.86–1.19
Others x Quartile 2	0.89	0.06	0.79–1.01
Others x Quartile 3	0.88	0.06	0.77–1.00
Others x Quartile 4	0.84	**0.03**	0.72–0.98
**Intimate partner violence**	5.77	<0.0001	5.01–6.65
**Insurance type**			
Private	Reference		
Medicare	1.94	<0.0001	1.88–2.01
Medicaid	1.51	<0.0001	1.45–1.57
Uninsured	3.63	<0.0001	3.46–3.80
Others	2.12	<0.0001	1.96–2.28

Compared to women aged 18–30 years, women aged 31–45 years had 1.23 times the odds of suicide/self-inflicted injury (OR = 1.23, p<0.0001, CI = 1.19–1.27) while women aged 46–65 years had 40% fewer odds of suicide/self-inflicted injury (OR = 0.61, p<0.001, CI = 0.59–0.63). Compared to Whites, Blacks (OR = 0.36, p<0.001, CI = 0.33–0.38) and Hispanics (OR = 0.34, p<0.001, CI = 0.30–0.39) had lower odds of suicide/self-inflicted injury. However, when we examined the interaction between race/ethnicity and income, Blacks in the highest income quartile had 20% increased odds of suicide/self-inflicted injury than Whites in the lowest income group (OR = 1.20, p = 0.01, CI = 1.05–1.37).

Other factors that predicted an increase in suicide/self-inflicted injury risk in the study were current smokers (OR = 2.33, p<0.001, CI = 2.24–2.43); intimate partner violence (OR = 5.77, p<0.001, CI = 5.01–6.65). With regards to insurance, participants with Medicare (OR = 1.94, p<0.001, CI = 1.88–2.01); Medicaid (OR = 1.51, p<0.001, CI = 1.45–1.57); and those who were uninsured (OR = 3.63, p<0.0001, CI = 3.46–3.80) had increased risk of self-inflicted injury compared to those with private insurance.

## Discussion

The study aimed to determine the factors associated with suicide/self-inflicted injury among females aged 18–65 years in the United States over thirteen years. Significant findings from the study are the association between age, race/ethnicity, smoking status, intimate partner violence, insurance type, and suicide/self-inflicted injury.

In this study, women aged 31–45 years had the highest suicide/self-inflicted injury prevalence. A previous study [33] reported that women aged 35–64 years had the highest suicide/self-inflicted injury prevalence, with a peak between 45 and 65 years. In this study, however, women aged 46–65 years had about 40% reduced odds of suicide/self-inflicted injury compared to women aged 18–30 years, like findings from other studies [34, 35]. Other studies have suggested that women’s suicide rate peaks at 35–44 years [36]. Changes in societal roles and mental health challenges could account for the increased risk for suicide/self-inflicted injury in this age group [35]. Among females aged 15–24 years, suicide is the leading cause of death globally [37], and the third leading cause of death in the U.S.; among those aged 25–34 years, suicide is the second leading cause of death [33]. Similarly, in this study, women ages 18–30 years had the second-highest proportion of suicide/self-inflicted injury. This finding implies that although suicide/self-inflicted injury prevention should be a universal intervention, younger women (women aged less than 45 years) are at higher risk of suicide/self-inflicted injury and should have more indicated suicide prevention interventions.

Blacks and Hispanics had a lower risk of suicide/self-inflicted injury in this study compared to Whites, like previous report [8]. Between 1999 and 2017, there has been an increase in suicide/self-inflicted injury rates across all racial-ethnic groups in the U.S., with the most significant increase seen among American Indians or Alaska Native [8]. Compared to Whites, all other racial/ethnic groups had a lower risk of suicide/self-inflicted injury in this study. Although studies have suggested a higher likelihood of suicide/self-inflicted injury misclassification among racial/ethnic minority individuals, an upward correction reduces the gap in suicide/self-inflicted injury rates between Blacks and Whites [38–40]. There is usually a more strongly negative connotation to suicide among racial/ethnic minorities. Therefore, the lower rates and risk of suicide may relate to the quality of ascertainment of death as suicide [41, 42]. It is highly like that an individual belonging to a racial/ethnic minority may be less likely to make the death easily recognized as suicide because of the impact of such determination on the family left behind. Other plausible explanations for less risk of suicide/self-inflicted injury among racial/ethnic minorities despite an increased risk include the influence of religion and a negative attitude towards suicide/self-inflicted injury [43, 44]. However, given that some studies have reported more suicide/self-inflicted injury attempts among African Americans than those in Whites [45], it is more likely that underreporting and misclassification of this condition account for the lower rates seen among African Americans and other racial/ethnic minorities [46–48].

In a sub-analysis, we explored the association between race/ethnicity, median income, and suicide/self-inflicted injury. Blacks in the highest income strata have a 20% increase in suicide/self-inflicted injury compared to Whites in the lowest income strata suggesting that the risk of suicide/self-inflicted injury among African-Americans is related to socioeconomic status, with higher income increases the risk. This finding is interesting because income as a variable is not an independent predictor of suicide/self-inflicted injury risk in the final regression model. In a previous study [49], being unfairly fired from work, being discouraged from moving into a neighborhood, being discouraged from education, and police abuse were associated with increased risk of suicidal ideation and suicide attempts. It is possible that Black women in the highest income strata are more likely to experience these forms of discriminations as they are more likely to interact with institutions that predominantly endorse a White-centric monocultural framework [50]. In addition, Blacks in the highest income bracket are likely to move into a different neighborhood that may be predominantly White, less connected to their communities, and less religious. These factors may increase their vulnerability to suicidal behaviors. Previous studies have shown that among Blacks, community belonginess and religion are protective factors against suicide [51, 52]. The feeling of ostracization, and repeated exposure to painful and fear-inducing situations increase vulnerability to suicidal ideation and suicidal behavior [50, 53]. Also, it is possible that there are racial differences in the associations between socioeconomic status and suicide [54, 55].

In this study, intimate partner violence increased the risk of suicide/self-inflicted injury 5-folds; other studies have reported similar associations [56–58]. In addition, studies have reported that posttraumatic stress disorder and depressive symptoms mediate the relationship between intimate partner violence and suicidal behavior [59]. Therefore, it is vital to assess for depressive symptoms and suicidal thoughts and behaviors among women who have experienced intimate partner violence.

Other risk factors for suicide/self-inflicted injury identified in this study include being a current smoker and uninsured or utilizing public insurance. A meta-analysis identified an increased risk of suicide, suicidal ideation among smokers compared to non-smokers [60]. Therefore, smoking cessation and smoking prevention programs directly impact suicide prevention and should be incorporated into suicide prevention programs. Having no insurance has also been shown to be a risk factor for suicide in another study [61].

### Limitation

There are several limitations to this study which needs to be taken into account in interpreting the results. First, the cross-sectional nature of the study design does not allow for causal inference, and assumes that many of the risk factors associated with suicidal behaviors are static. Secondly, using the ICD E-codes although has 83–100% positive predictive value, but a sensitivity of 2–19% [32], therefore, there is high possibility that the number of cases identified vastly underestimates the true number of cases of suicide and self-inflicted injuries. In addition to undercoding, some cases of self-inflicted injuries are accidental and not suicidal; their inclusion in the analysis may have overstated the estimate of suicidal behaviors in this study. However, non-suicidal self-inflicted injuries cannot be said to be entirely non-suicidal: studies have found co-existing suicidal ideation and behaviors in people with accidental (non-suicidal) self-inflicted injuries, subsequent suicidal thoughts and behaviors are higher among these individual, and accidental self-inflicted injuries longitudinally predicts subsequent suicidal behaviors [62–64]. Thirdly, there is a general under-utilization of code modifiers for intimate partner violence which may affect the estimate of the relationship between intimate partner violence and suicide found in this study [65, 66]. Fourthly, we did not include mental health disorders and substance use disorders in our analysis. Several studies have reported association between depression, anxiety, personality disorders, substance use disorders and suicide, with a much higher relative risk of suicide in women with substance use disorders [13, 67–70]. However, majority of people who die by suicide have never seen a mental health provider or ever had a diagnosis of mental health disorder [71, 72]. While it is important to identify women with mental illness and substance use disorder as these individuals represent high risk individuals, suicide prevention efforts need to focus on identifying other universal risk factors for suicidal behaviors [23]. Early intervention to prevent suicide attempts and suicidal behaviors may warrant shifting the focus from individual factors to ecological factors such as social, economic, and cultural factors, including prevention of intimate partner violence [10, 73].

## Conclusions

This study showed that among adult women in the United States, the highest risk of suicide/self-inflicted injury is among women aged 31–45 years, higher earning Black women, women who have experienced intimate partner violence, uninsured, and current smokers. In addition to universal suicide prevention strategies such as universal screening and suicide hotlines, interventions and policies that reduce smoking, prevents intimate partner violence, addresses racial discrimination and bias, and provides universal health coverage are needed to prevent excess mortality from suicide deaths.
