# Correction: Factors associated with suicide/self-inflicted injuries among women aged 18–65 years in the United States: A 13-year retrospective analysis of the National Inpatient Sample database

**DOI:** 10.1371/journal.pone.0316922

**Published:** 2024-12-31

**Authors:** Oluwasegun Akinyemi, Temitope Ogundare, Adeolu Funso Oladunjoye, Kindha Elleissy Nasef, Christina Lipscombe, John Akinshola Akinbote, Maureen Bezold

The third author’s name is spelled incorrectly. The correct name is: Adeolu Funso Oladunjoye.

The affiliation for the third author is incorrect. The correct affiliation is not indicated. Adeolu Funso Oladunjoye is not affiliated with #4 but with: Department of Psychiatry, Baylor College of Medicine, Houston, TX, United States of America.
